# Cystatin F involvement in adenosine A_2A_ receptor-mediated neuroinflammation in BV2 microglial cells

**DOI:** 10.1038/s41598-018-25031-5

**Published:** 2018-05-01

**Authors:** Wei Duan, Haoxiang Wang, Qinlin Fan, Lin Chen, Heqing Huang, Hong Ran

**Affiliations:** 1 Department of Neurology, Xinqiao Hospital, Third Military Medical University, 2-V Xinqiao Street, Shapingba District, Chongqing, 400037 China; 2 Department of Neurology, Southwest Hospital, Third Military Medical University, 30 Gaotanyan Street, Shapingba District, Chongqing, 400038 China; 3 Administration Center of Clinical Education, Southwest Hospital, Third Military Medical University, 30 Gaotanyan Street, Shapingba District, Chongqing, 400038 China

## Abstract

Our previous studies have shown adenosine A_2A_ R activation markedly promotes the expression of cystatin F (CF) and exacerbates the white matter lesions induced by hypoxic brain injuries. Thus, we hypothesized that CF was probably involved in neuroinflammation of activated microglia induced by A_2A_ R activation. We transfected the BV2 cells with a CF shRNA vector and examined the production of pro-inflammatory cytokines in hypoxic-BV2 cells in which A_2A_ R was activated or inactivated to confirm this hypothesis. Additionally, we also investigated the probable signaling pathways involved in modulation of A_2A_ R activation on CF expression in hypoxia-activated BV2 cells. Activation of A_2A_ R promoted CF expression, which was significantly increased after the low glucose and hypoxia treatments in BV2 cells. CF gene knockdown markedly inhibited the increase in the expression of pro-inflammatory cytokines induced by A_2A_ R activation in hypoxic-BV2 cells. Furthermore, the increased expression of the CF induced by A_2A_ R activation was remarkably inhibited in hypoxic-BV2 cells administrated with the PKA inhibitor H-89 and the PKC inhibitor staurosporine. Hence, these results indicate that hypoxia BV2 cells highly express CF, which is involved in A_2A_ R activation-mediated neuroinflammation via the PKA/CREB and PKC/CREB or ERK1/2 signaling pathways.

## Introduction

Adenosine, which activates the A_2A_ receptor (A_2A_ R), has been reported to play an important role in regulating the inflammatory response in various types of brain injuries^1–3^. A_2A_ R knockout has been shown to protect against acute ischemic brain injury by reducing glutamate outflow and decreasing pro-inflammatory cytokine production^4,5^. However, the underlying mechanisms of the modulation of the inflammatory response in acute ischemic brain injury via A_2A_ R knockout have remained elusive. Contrary to the effect of A_2A_ R knock-out on the acute cerebral ischemia, our data indicate that A_2A_ R gene knockout aggravates white matter rarefaction, promotes microglial activation and increases pro-inflammatory cytokines expression in white matter lesions in a mouse model of chronic cerebral hypoperfusion^6^. However, the signaling pathways involved in the effects of the A_2A_ R on inflammatory cytokines production during chronic ischemic brain damage are not known. An understanding of the mechanisms by which A_2A_ R modulates the neuroinflammatory response in various types of ischemic brain injury may indicate the therapeutic potential of this receptor.

A recent study indicated that cystatin F (CF), a potent endogenous cysteine protease inhibitor, is primarily expressed in activated microglia in the diseased central nervous system; however, it is not expressed in the normal brain^7^. More importantly, CF expression was substantially up-regulated in regions of white matter rarefaction that occurred in various demyelinating diseases of the central nervous system^7,8^. Consistent with these findings, our previous study showed that CF was expressed in activated microglial cells in white matter lesions induced by chronic cerebral hypoperfusion. Importantly, A_2A_ R inactivation substantially increased CF production in activated microglial cells; moreover, the production of inflammatory cytokines was significantly increased in white matter lesions after chronic cerebral hypoperfusion^9^. These results suggested that CF expressed in activated microglia may be associated with the A_2A_ R effect on the neuroinflammatory response induced by chronic cerebral hypoperfusion. CF is an inhibitor of the papain-like C1 family of cysteine proteases, which includes endosomal/lysosomal cathepsins^10^, and is predominantly expressed in immune cells and tissues, such as monocytes, dendritic cells and certain types of T cells and natural killer (NK) cells^11,12^. Numerous studies have recently indicated that CF exerts a pro-inflammatory role via the increased production of active pro-inflammatory cytokines during inflammatory responses^13,14^ and the regulation of immune cell differentiation and maturation^11^. Accordingly, these results suggest that CF produced by activated microglial cells may substantially enhance the expression of pro-inflammatory cytokines and exacerbate inflammatory damage to the central nervous system. Based on our previous findings, we hypothesize that CF expressed in activated microglial cells may be strongly associated with the impacts of A_2A_ R activation on neuroinflammation in white matter lesions induced by chronic cerebral hypoperfusion. However, the underlying signaling pathways involved in the A_2A_ R effect on CF expression in activated microglia are unknown.

The immortalized murine microglial cell line BV2, which has a reaction pattern with many similarities to primary microglia, has frequently been employed as a substitute for primary microglia^15^. Here, we transfected BV2 cells with CF-targeting short hairpin RNA (shRNA) viral vectors and control vector, exposed the cells to low glucose and hypoxia, activated or inactivated A_2A_ R in hypoxic BV2 cells with a selective agonist or antagonist, respectively, and examined the expression of CF and the production of pro-inflammatory cytokines, including TNF-α, IL-1β and IL-6, via quantitative real-time reverse transcription-PCR (qRT-PCR), western blot analyses and ELISAs. Moreover, we investigated the effects of the probable signaling pathways involved in modulating A_2A_ R activation on CF expression in low glucose- and hypoxia-activated BV2 cells via qRT-PCR, western blot analyses and immunofluorescence staining.

## Results

### Detection of cell viability and examination of the silencing efficiency of CF in BV2 cells

**Figure 1:**
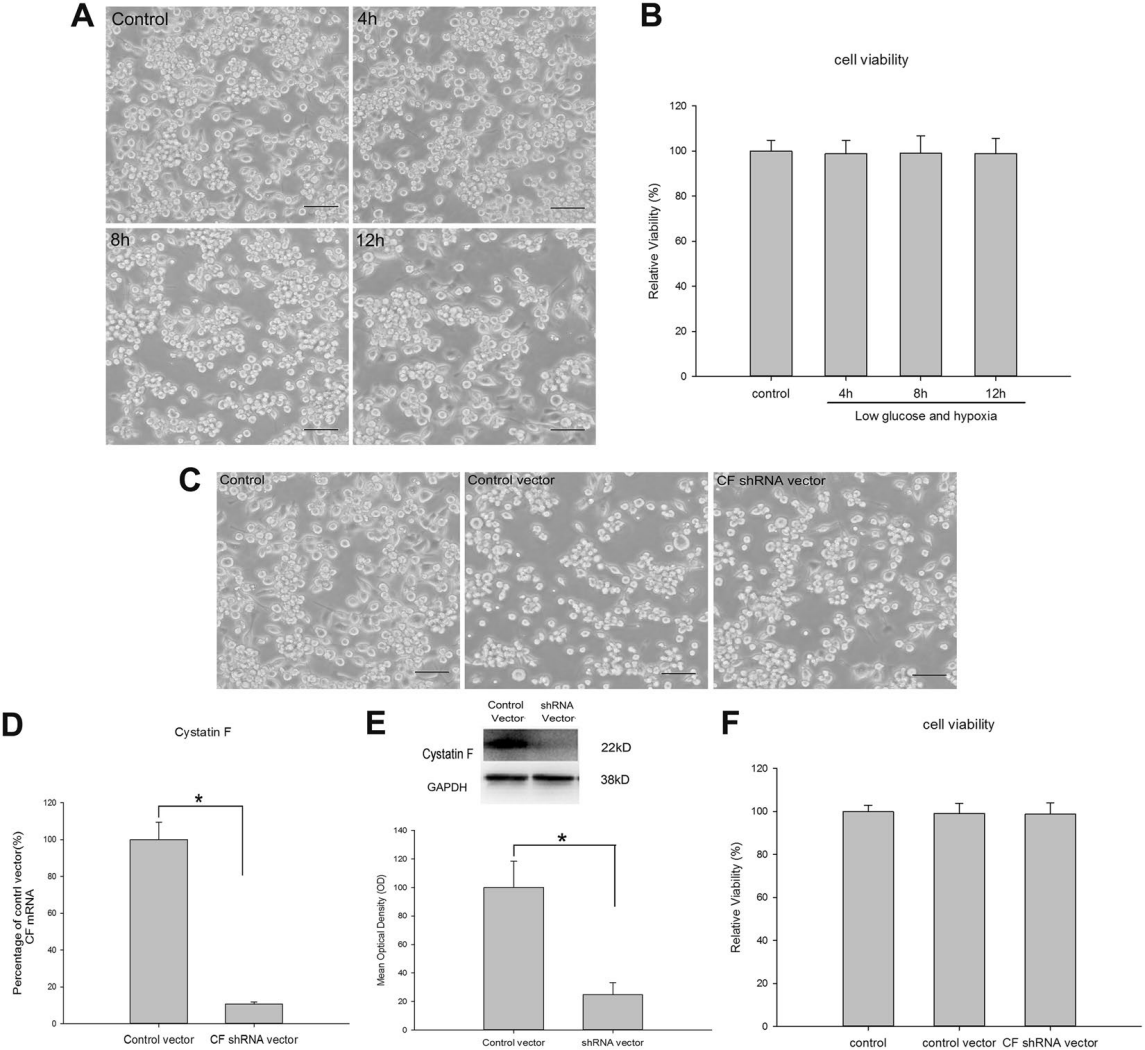
Examination of cell viability of BV2 cells after low glucose and hypoxia or transfected with vectors and detection of the silencing efficiency of CF in BV2 cells. The external cell morphology appears relatively unchanged in BV2 cells exposed to low glucose and hypoxia for 4 h, 8 h and 12 h when compared with the control (**A**). Bar graph shows the cell viability of BV2 cells after low glucose and hypoxia compared to control (**B**). Note that there was no significantly difference in the cells viability of cells in every group (**B**). The external cell morphology appears relatively unchanged in BV2 cells transfected with control vector or CF shRNA vector when compared with the control (**C**). Bar graph in the (**F**) shows the cell viability of BV2 cells transfected with control vector or CF shRNA vector when compared with the control. There was no significantly difference in the cells viability of BV2 cells in every group (**F**). The silencing efficiency of CF shRNA vector were analyzed by RT-PCR (**D**), western blots (**E**). RT-PCR analysis shows that the efficiency of shRNA vector-mediated suppression of CF expression is about 89.27% when compared to control vector cells (**D**). The upper panel of (**E**) shows the specific band of CF (20 kDa) and GAPDH (38 kD). Bar graph in the lower panel of (**E**) shows significant change in the mean optical density of CF protein expression between control vector cells and shRNA vector cells. The protein expression of CF in shRNA vector cells significantly reduced compared with that in control vector cells. *p < 0.05. The values represent the mean ± SEM in triplicate. Scale bars = 100 μm (**A** and **C**).

At 4 h, 8 h and 12 h after the low glucose and hypoxia treatments, the cell viability in each group was assessed using the tetrazolium salt-based colorimetric assay from the Cell Counting Kit 8 (CCK-8). There was no marked difference in the cell viability among the groups at 4 h, 8 h and 12 h after the low glucose and hypoxia treatments (Fig. 1B).

We used an shRNA vector to knockdown the expression of the CF gene in BV2 cells and further investigate the role of CF in the modulation of inflammation in response to A_2A_ R activation or inactivation in hypoxic BV2 cells. BV2 cells were transfected with the CF shRNA vector or a control vector. The CF mRNA levels in the BV2 cells that had been transfected with the CF shRNA vector were significantly decreased compared with the levels in the BV2 cells that had been transfected with the control vector (*p* < 0.05, Fig. 1D). The silencing efficiency was approximately 89.27% compared to that of the control vector-transfected BV2 cells. Moreover, the CF protein levels were significantly reduced in the CF shRNA-transfected BV2 cells compared to the levels in the control vector-transfected BV2 cells, as indicated by western blot analysis (*p* < 0.05, Fig. 1E). Furthermore, the viability of the CF shRNA vector or control vector-transfected cells was detected using CCK-8. There was no significant difference in the cell viability between the shRNA vector-transfected cells and control cells or the control vector-transfected cells and control cells (Fig. 1F).

### The mRNA level and protein expression of CF in hypoxic-BV2 cells significantly increased after activation of adenosine A_2A_ receptors

**Figure 2:**
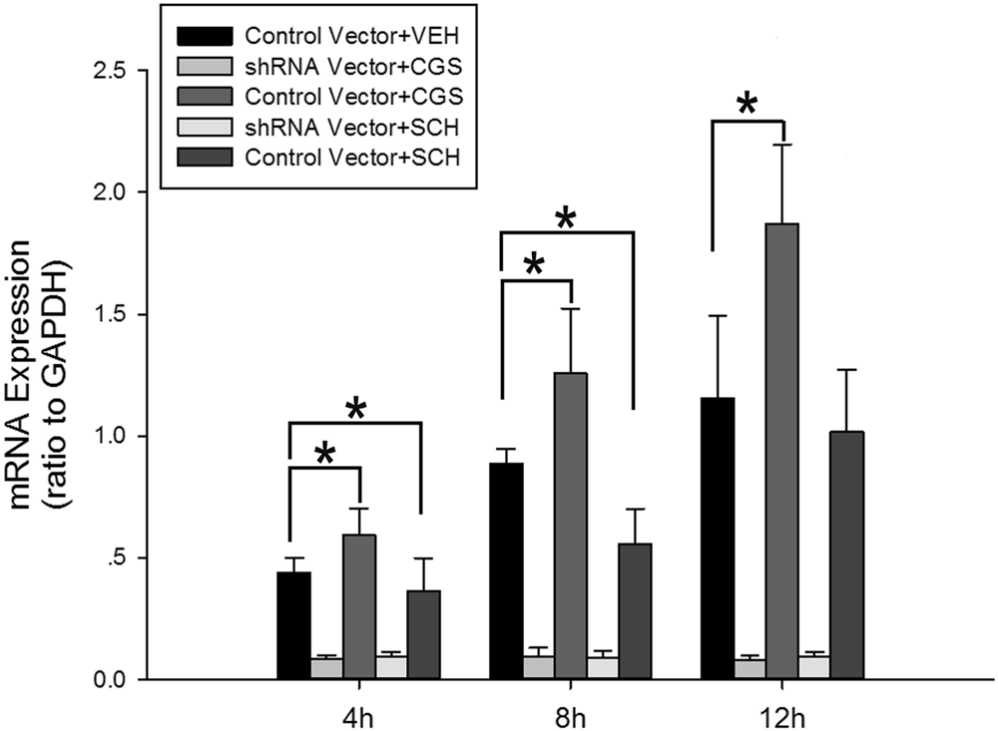
qRT-PCR analysis of CF mRNA level in hypoxic-BV2 cells with A_2A_ R activation by CGS21680 or A_2A_ R inactivation by SCH58261 after 4 h, 8 h and 12 h of exposure to low glucose and hypoxia. Compared with control vector + VEH group, the mRNA level of CF in BV2 cells with A_2A_ R activation by CGS21680 significantly increased, whereas the mRNA level of CF in BV2 cells with A_2A_ R inactivation by SCH58261 markedly reduced after 4 h and 8 h of exposure to low glucose and hypoxia. *p < 0.05. The values represent the mean ± SEM in triplicate.

**Figure 3:**
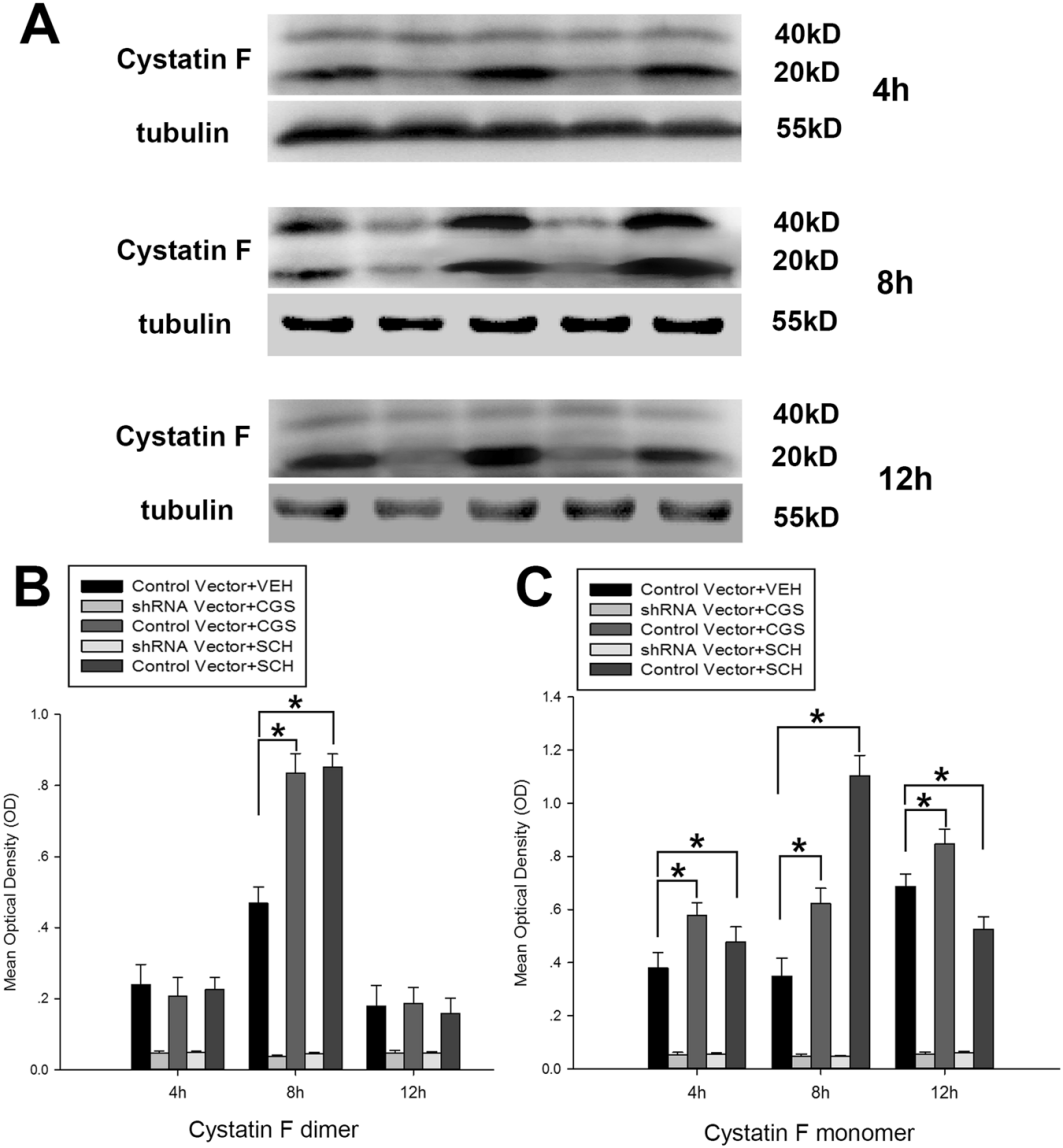
Western blot analysis of CF protein expression in hypoxic-BV2 cells with A_2A_ R activation by CGS21680 or A_2A_ R inactivation by SCH58261 after 4 h, 8 h and 12 h of exposure to low glucose and hypoxia. The upper panel shows the specific band of CF monomer (20 kD) and CF dimer (40 kD) in 4 h, 8 h and 12 h after exposure to low glucose and hypoxia. The bar graphs in the lower panel of Fig. 3 shows significant changes in the mean optical density of CF dimer (**B**) and CF monomer (**C**) in BV2 cells with A_2A_ R activation or A_2A_ R inactivation after 4 h, 8 h and 12 h of exposure to low glucose and hypoxia. The protein expression of CF monomer in control vector + CGS group significantly increased, whereas the protein expression of CF monomer in control vector + SCH group significantly reduced after 4 h and 12 h of exposure to low glucose and hypoxia when compared with control vector + VEH group. Compared with control vector + VEH group, the protein expression of CF dimer in control vector + CGS group significantly increased at 8 h after exposing to low glucose and hypoxia. However, the protein expression of CF dimer in control vector + SCH group also significantly increased after 8 h of exposure to low glucose and hypoxia when compared with control vector + VEH group. *p < 0.05. The values represent the mean ± SEM in triplicate.

The selective A_2A_ R agonist CGS21680 and the selective A_2A_ R antagonist SCH58261 were added to cells 10 min prior to the low glucose and hypoxia treatments to assess changes in CF expression in response to adenosine A_2A_ R activation or inactivation. The CF mRNA and protein levels in the BV2 cells were subsequently examined at 4 h and 8 h after the cells were exposed to low glucose and hypoxia using qRT-PCR and western blotting. Figure 2 shows the comparison of the CF mRNA levels in the A_2A_ R-activated BV2 cells or A_2A_ R-inactivated BV2 cells at 4 h, 8 h and 12 h after the cells were exposed to low glucose and hypoxia. Following the addition of the drug, CGS21680 significantly increased the CF mRNA levels at 4 h, 8 h and 12 h after the cells were exposed to low glucose and hypoxia (*p* < 0.05, Fig. 2). In contrast, SCH 58261 significantly reduced the CF mRNA levels at 4 h and 8 h after the low glucose and hypoxia treatments (*p* < 0.05, Fig. 2). The protein expression of the CF dimer and CF monomer in the A_2A_ R-activated BV2 cells or A_2A_ R-inactivated BV2 cells was measured by western blotting at 4 h, 8 h and 12 h after the cells were exposed to low glucose and hypoxia (Fig. 3). Consistent with the mRNA level analysis, CGS21680 substantially increased the protein levels of the CF monomer at 4 h, 8 h and 12 h after the cells were exposed to low glucose and hypoxia (*p* < 0.05, Fig. 3C) and the level of CF dimer at 8 h after the cells were exposed to low glucose and hypoxia (*p* < 0.05, Fig. 3B). In contrast, SCH58261 significantly inhibited the protein levels of CF monomer in BV2 cells at 4 h and 12 h after the cells were exposed to low glucose and hypoxia (*p* < 0.05, Fig. 3C), whereas it increased the protein levels of CF dimer and CF monomer in BV2 cells at 8 h after the cells were exposed to low glucose and hypoxia (*p* < 0.05, Fig. 3B and C).

### Knockdown of CF expression in hypoxic-BV2 cells inhibited the production of pro-inflammatory mediators afforded by activation or inactivation of adenosine A_2A_ receptors

**Figure 4:**
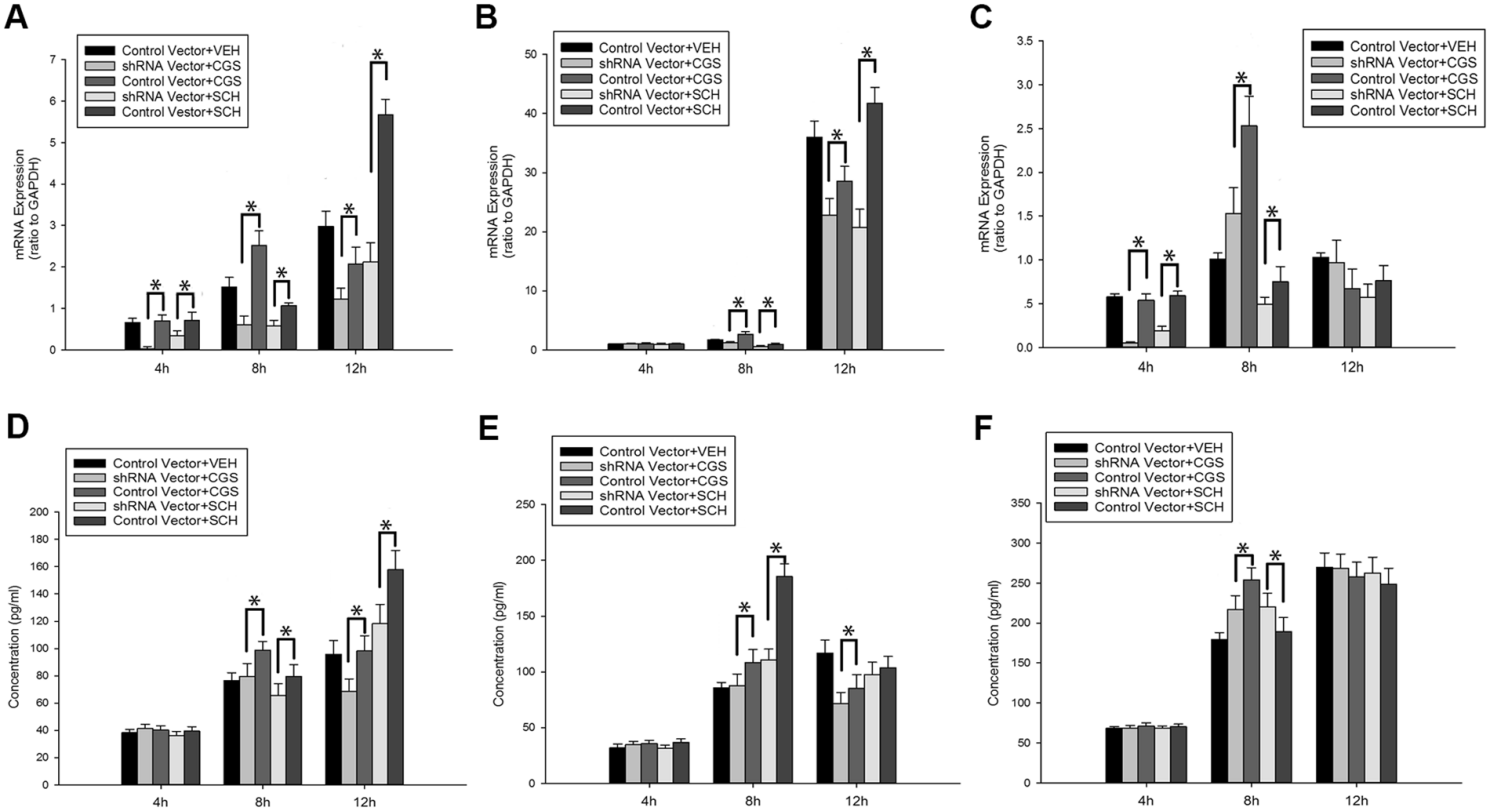
The mRNA and protein levels of pro-inflammatory cytokines in control vector transfected cells and CF shRNA vector transfected cells treated with A_2A_ R activation by CGS21680 or A_2A_ R inactivation by SCH58261 after 4 h, 8 h and 12 h of exposure to low glucose and hypoxia. The mRNA levels of IL-1β (**A**), IL-6 (**B**) and TNF-α (**C**) in control vector transfected BV2 cells and CF shRNA vector transfected BV2 cells treated with A_2A_ R activation by CGS21680 or A_2A_ R inactivation by SCH58261 were detected by qRT-PCR after 4 h, 8 h and 12 h of exposure to low glucose and hypoxia. (**D**–**F**) showed the ELISA analysis of protein expressions of IL-1β (**D**), IL-6 (**E**) and TNF-α (**F**) in control vector transfected cells and CF shRNA vector transfected cells treated with A_2A_ R activation by CGS21680 or A_2A_ R inactivation by SCH58261 after 4 h, 8 h and 12 h of exposure to low glucose and hypoxia. *p < 0.05. The values represent the mean ± SEM in triplicate.

We further investigated the effect of CF gene knockdown on the expression of IL-1β, IL-6 and TNF-α in hypoxic BV2 cells following A_2A_ R activation or inactivation at 4 h, 8 h and 12 h after the cells were exposed to low glucose and hypoxia via quantitative PCR. Figure 4(A–C) presents the comparison of the IL-1β, IL-6 and TNF-α mRNA levels in the CF shRNA vector-transfected BV2 cells and control vector-transfected BV2 cells following A_2A_ R activation with CGS21680 or A_2A_ R inactivation with SCH58261 at 4 h, 8 h and 12 h after the cells were exposed to low glucose and hypoxia. The IL-1β mRNA levels in the shRNA vector + CGS/SCH group were significantly decreased compared with those in the control vector + CGS/SCH group at 4 h, 8 h and 12 h after the cells were exposed to low glucose and hypoxia (*p* < 0.05, Fig. 4A). Similarly, the IL-6 mRNA levels in the shRNA vector + CGS/SCH group were significantly decreased compared with those in the control vector + CGS/SCH group at 8 h and 12 h after the cells were exposed to low glucose and hypoxia (*p* < 0.05, Fig. 4B). The TNF-α mRNA levels in the shRNA vector + CGS/SCH group were significantly decreased compared with those in the control vector + CGS/SCH group at 4 h and 8 h after the cells were exposed to low glucose and hypoxia (*p* < 0.05, Fig. 4C). However, there were no differences in the IL-6 mRNA levels between these two groups 4 h after exposure to low glucose and hypoxia (Fig. 4B). Moreover, there was no difference in the TNF-α mRNA levels between the shRNA vector + CGS/SCH group and control vector + CGS/SCH group at 12 h after the cells were exposed to low glucose and hypoxia (Fig. 4C).

In addition, the effects of CF gene knockdown on the protein secretion of IL-1β, IL-6 and TNF-α in hypoxic BV2 cells in response to A_2A_ R activation or inactivation were measured by ELISA at 4 h, 8 h and 12 h after the cells were exposed to low glucose and hypoxia. The protein secretion of IL-1β, IL-6 and TNF-α in the CF shRNA vector-transfected BV2 cells significantly decreased compared with the that in control vector-transfected BV2 cells after exposure to low glucose and hypoxia (*p* < 0.05, Fig. 4D–F). The secretion of IL-1β protein in the shRNA vector + CGS/SCH group was significantly decreased compared with that in the control vector + CGS/SCH group at 8 h and 12 h after the cells were exposed to low glucose and hypoxia (*p* < 0.05, Fig. 4D). Moreover, the secretion of IL-6 in the shRNA vector + CGS/SCH group was significantly decreased compared with that in the control vector + CGS/SCH group at 8 h after the cells were exposed to low glucose and hypoxia (*p* < 0.05, Fig. 4E). The secretion of IL-6 in the shRNA vector + CGS group was significantly decreased compared with that in the control vector + CGS group at 12 h after the cells were exposed to low glucose and hypoxia (*p* < 0.05, Fig. 4E). The secretion of TNF-α protein in the shRNA vector + CGS group was significantly decreased compared with that in the control vector + CGS group at 8 h after the cells were exposed to low glucose and hypoxia (*p* < 0.05, Fig. 4F). However, there were no differences in the secretion of TNF-α protein between the CF shRNA viral vector-transfected BV2 cells and the control vector-transfected BV2 cells at 4 h and12 h after the cells were exposed to low glucose and hypoxia (Fig. 4F).

**Figure 5:**
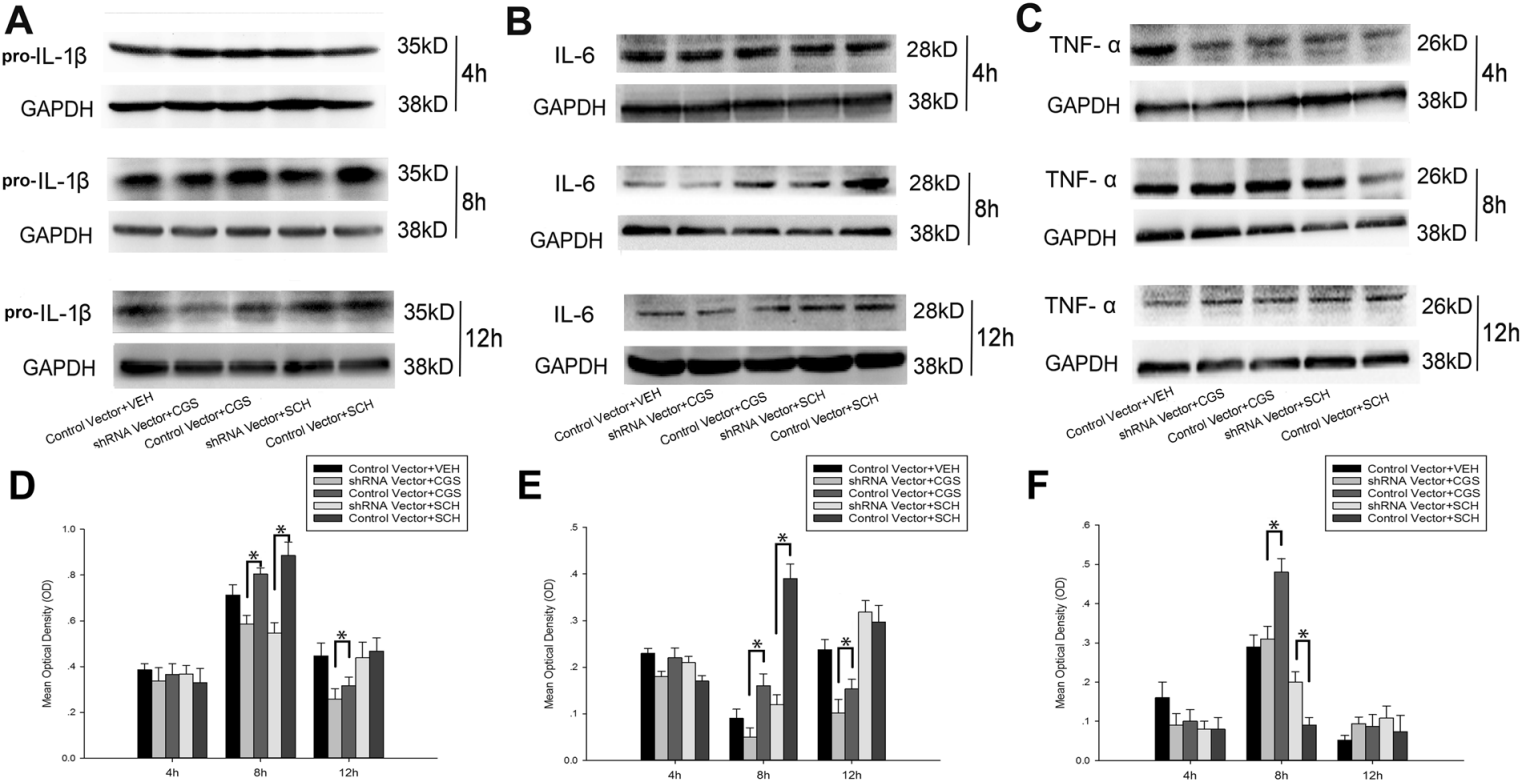
Western blot analysis of protein expressions of pro-inflammatory cytokines in control vector transfected cells and CF shRNA vector transfected cells treated with A_2A_ R activation by CGS21680 or A_2A_ R inactivation by SCH58261 after 4 h, 8 h and 12 h of exposure to low glucose and hypoxia. The upper panel shows the specific band of pro-IL-1β (**A**), IL-6 (**B**) and TNF-α (**C**) after 4 h, 8 h and 12 h of exposure to low glucose and hypoxia. The lower panel shows bar graphs showing significant changes in mean optical density of pro-IL-1β (**D**), IL-6 (**E**) and TNF-α (**F**) in each group after 4 h, 8 h and 12 h of exposure to low glucose and hypoxia. At 8 h after exposure to low glucose and hypoxia, the protein expression of pro-IL-1β, IL-6 and TNF-α in shRNA vector + CGS group significantly reduced when compared with that in control vector + VEH group. *p < 0.05. The values represent the mean ± SEM in triplicate.

Consistent with the ELISA analysis results, the pro-IL-1β, IL-6 and TNF-α protein levels detected by western blot were significantly reduced in the shRNA vector + CGS group and the shRNA vector + SCH group compared with those in the control vector + CGS group and the control vector + SCH group at 8 h after the cells were exposed to low glucose and hypoxia (*p* < 0.05, Fig. 5A–C). Moreover, the protein levels of pro-IL-1β and IL-6 were substantially reduced in the shRNA vector + CGS group compared with those in the control vector + CGS group at 12 h after the cells were exposed to low glucose and hypoxia (*p* < 0.05, Fig. 5A and B). However, there was no difference in the TNF-α secretion between the two groups at 12 h after the cells were exposed to low glucose and hypoxia (Fig. 5C).

### PKA-CREB and PKC-ERK1/2 signaling pathways are associated with the effect of adenosine A_2A_ receptor activation on the expression of CF in hypoxic-BV2 cells after low glucose and hypoxia treatment

**Figure 6:**
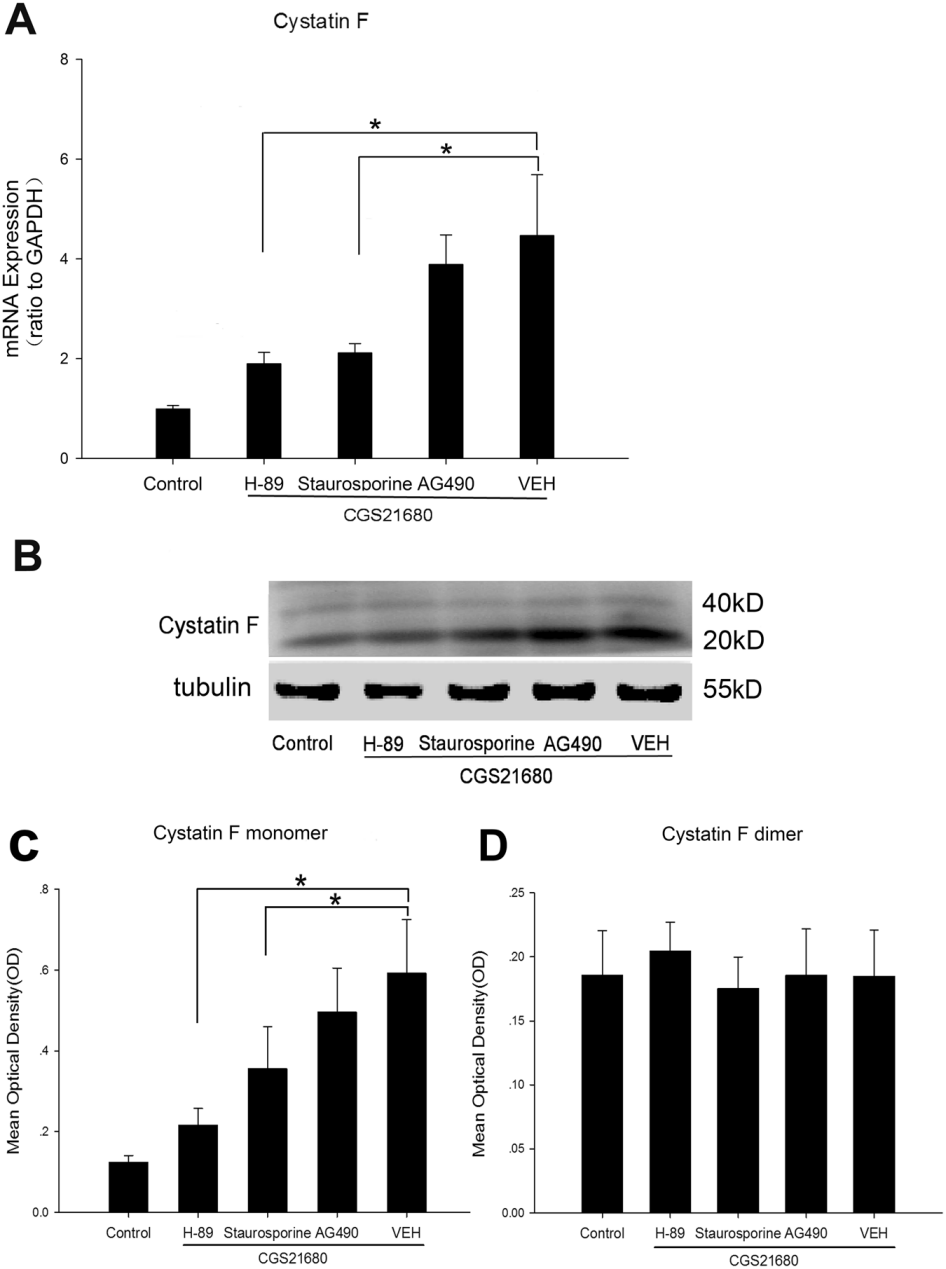
qRT-PCR and western blot analysis of CF expression in hypoxic-BV2 cells administrated with H-89, Staurosporine and AG490 before A_2A_ R activation by CGS21680 after 8 h of exposure to low glucose and hypoxia. The bar graph shows significantly changes in the mRNA levels of CF in BV2 cells administrated with H-89, Staurosporine and AG490 before A2A R activation by CGS21680 after 8 h of exposure to low glucose and hypoxia (**A**). The mRNA levels of CF in H-89 group, Staurosporine group, AG490 group and VEH group which were administrated with CGS21680 significantly increased when compared with control group after 8 h of exposure to low glucose and hypoxia. The mRNA levels of CF in H-89 group and Staurosporine group remarkably decreased when compared with VEH group after 8 h of exposure to low glucose and hypoxia. The (**B**) shows the specific band of CF dimer (40 kD) and CF monomer (20 kD) in every group. The bar graphs in (**C** and **D**) showed significantly changes in the protein expression of CF dimer (**C**) and CF monomer (**D**) in BV2 cells administrated with H-89, Staurosporine and AG490 before A_2A_ R activation by CGS21680 after 8 h of exposure to low glucose and hypoxia. The protein expression of CF monomer in H-89 group and Staurosporine group significantly decreased when compared with VEH group after 8 h of exposure to low glucose and hypoxia. There were no significantly differences in the protein expression of CF dimer in every group after 8 h of exposure to low glucose and hypoxia. *p < 0.05. The values represent the mean ± SEM in triplicate.

Hypoxic BV2 cells were treated with the PKA inhibitor H-89, the PKC inhibitor staurosporine and the JAK inhibitor AG490 prior to the administration of CGS21680; the CF mRNA and protein levels were subsequently determined by quantitative PCR, western blot and immunofluorescence staining at 8 h after the cells were exposed to low glucose and hypoxia to further explore the signaling pathway involved in the effects of A_2A_ R activation on CF expression in BV2 cells. The CF mRNA levels in the H-89, staurosporine, AG490 and VEH groups, which were all treated with CGS21680, were significantly increased compared with those in the control group at 8 h after the cells were exposed to low glucose and hypoxia (*p* < 0.05, Fig. 6A). Moreover, the CF mRNA levels in the H-89 and staurosporine groups were substantially decreased compared with those in the VEH group after A_2A_ R activation with CGS21680 at 8 h after the cells were exposed to low glucose and hypoxia (*p* < 0.05, Fig. 6A). There was no difference in the CF mRNA levels between the AG490 and VEH groups after A_2A_ R activation at 8 h after the cells were exposed to low glucose and hypoxia.

**Figure 7:**
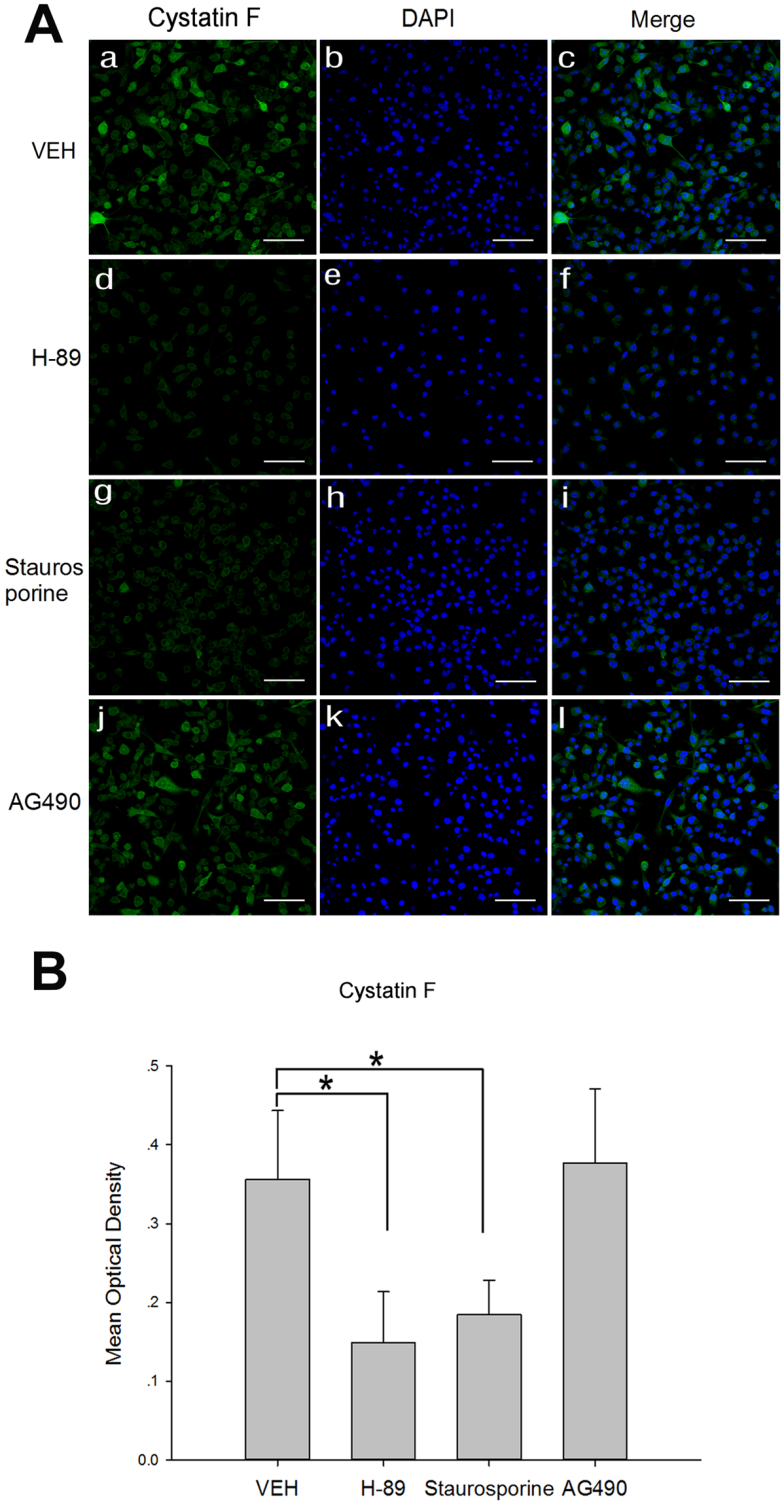
Immunofluorescence staining for CF in H-89 group, Staurosporine group and AG490 group after administrated with CGS21680 after 8 h of exposure to low glucose and hypoxia. (**A**) Immunofluorescence images of CF in each group. (**B**) The bar graphs showing significant changes in mean optical density of CF immunoreactivity in each group. The immunofluorescence density of CF in the H-89 group and Staurosporine group significantly decreased when compared with VEH group after 8 h of exposure to low glucose and hypoxia. *p < 0.05. The values represent the mean ± SEM in triplicate. Scale bar in A = 30 μm.

**Figure 8:**
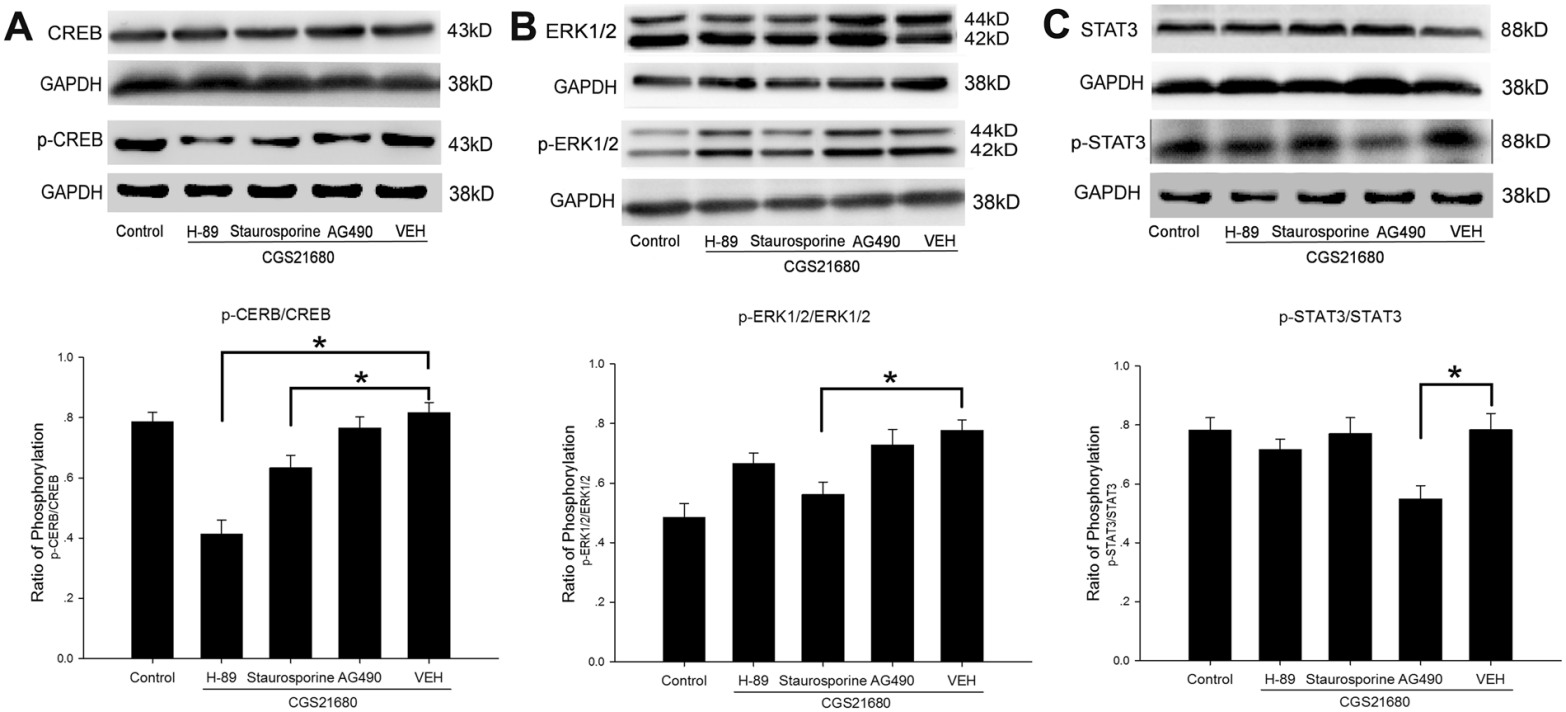
Western blot analysis of the phosphorylated substrate proteins in signal pathways in hypoxic-BV2 cells after administrated with CGS21680 after 8 h of exposure to low glucose and hypoxia. The upper panel shows the specific band of CREB and p-CREB (43 kD, **A**), ERK1/2 and p-ERK1/2 (44 kD/42 Kd, **B**), STAT3 and p-STAT3 (88 Kd, **C**) and GAPDH (38 kD). The lower panel shows bar graphs showing significant changes in the optical density ratio of phosphor-proteins to that of total proteins between groups after 8 h of exposure to low glucose and hypoxia. The ratio of phosphorylation of CREB in H-89 group and Staurosporine group significantly decreased when compared with VEH group after 8 h of exposure to low glucose and hypoxia. The ratio of phosphorylation of ERK1/2 in Staurosporine group also significantly decreased when compared with VEH group after 8 h of exposure to low glucose and hypoxia. *p < 0.05. The values represent the mean ± SEM in triplicate.

In addition, the expression of CF protein (Fig. 6B–D) and the phosphorylated substrate proteins in the PKA, PKC and JAK pathways (Fig. 8) in each CGS21680-treated group were examined via western blot at 8 h after the cells were exposed to low glucose and hypoxia. The protein expression of the CF monomer was significantly decreased in the H-89 and staurosporine groups compared with that in the VEH group at 8 h after the cells were exposed to low glucose and hypoxia (*p* < 0.05, Fig. 6C). However, there was no significant difference in the protein expression of the CF monomer between the BV2 cells in the AG490 and VEH groups at 8 h after the cells were exposed to low glucose and hypoxia (Fig. 6C). There was no significant difference in the protein levels of CF dimer among the groups at 8 h after the cells were exposed to low glucose and hypoxia (Fig. 6D). The protein expression of CF in the H-89, staurosporine and AG490 groups was also assessed by immunofluorescence staining to further confirm the role of CF in BV2 cells with A_2A_ R activation following low glucose and hypoxia treatments (Fig. 7). Consistent with the western blot analysis results for the CF protein expression, the density of the CF immunofluorescence was significantly decreased in the H-89 and staurosporine groups compared with that in the VEH group at 8 h after the cells were exposed to low glucose and hypoxia (*p* < 0.05, Fig. 7B). However, there was no difference in the density of the CF immunofluorescence between the VEH and AG490 groups 8 h after the cells were exposed to low glucose and hypoxia (Fig. 7B).

Figure 8 presents the protein expressions of CREB and p-CREB (Fig. 8A), ERK1/2 and p-ERK1/2 (Fig. 8B), and STAT3 and p-STAT3 (Fig. 8C) in hypoxic BV2 cells treated with H-89, staurosporine and AG490 prior to A_2A_ R activation with CGS21680 at 8 h after the cells were exposed to low glucose and hypoxia. Compared with the VEH group, the H-89 and staurosporine groups showed significantly decreased ratios of the mean optical density of p-CREB and CREB 8 h after the cells were exposed to low glucose and hypoxia as detected by western blot (*p* < 0.05, Fig. 8A). Compared with the VEH group, the staurosporine group showed a significantly decreased ratio of the mean optical density of p-ERK1/2 and ERK1/2 8 h after the cells were exposed to low glucose and hypoxia as detected by western blot (*p* < 0.05, Fig. 8B). Furthermore, the ratio of the mean optical density of p-STAT3 and STAT3 detected by western blot was substantially reduced in the AG490 group compared with that in the VEH group at 8 h after the cells were exposed to low glucose and hypoxia (*p* < 0.05, Fig. 8C).

## Discussion

In the present study, the adenosine A_2A_ R was selectively activated in cultured hypoxic BV2 cells following treatment with CGS21680 and was inactivated after treatment with SCH58261. We subsequently examined the mRNA and protein levels of CF and pro-inflammatory cytokines in the hypoxic BV2 cells using quantitative PCR and western blot analyses after the low glucose and hypoxia treatments. We showed that A_2A_ R activation by CGS21680 promoted CF expression, which was significantly increased after the low glucose and hypoxia treatments. More importantly, we inhibited CF expression in BV2 cells by transfecting the cells with a CF shRNA viral vector and showed that the increased expression of CF may be involved in the effects of A_2A_ R activation on the increased production of pro-inflammatory cytokines in hypoxic BV2 cells in response to the low glucose and hypoxia treatments. Furthermore, our results indicated that A_2A_ R activation regulated CF expression in hypoxic BV2 cells through the PKA-CREB and PKC-CREB/ERK1/2 signaling pathways.

It is well established that adenosine A_2A_ R activation plays an important role in modulating inflammation in a broad spectrum of brain injuries, including ischemic and hemorrhagic brain injuries^16–18^. Microglial cells are the primary inflammatory cells in the central nervous system and are involved in neuroinflammation associated with various brain insults via pro-inflammatory cytokine expression^19,20^. Moreover, activated microglial cells are associated with the complex regulatory effect of A_2A_ R activation on the neuroinflammatory response in brain injuries. Our previous *in vivo* experiment indicated that A_2A_ R knockout exacerbated the white matter lesions induced by chronic cerebral hypoperfusion by promoting microglial cell activation and increasing pro-inflammatory cytokine expression^6^. However, previous studies have suggested that A_2A_ R inactivation suppressed microglial activation and nitric oxide release in cultured microglial cells exposed to an inflammatory stimulus, such as lipopolysaccharide (LPS)^21,22^. Thus, adenosine A_2A_ R activation exerts a complex effect on microglial activation and the expression of inflammatory cytokines in neuroinflammation. It remains unclear how A_2A_ R activation exerts its important and complex modulation of neuroinflammation in various brain injuries. Numerous results have shown that the regulation of pro-inflammatory cytokine expression and the activation of microglia cells are involved in the A_2A_ R-mediated modulation of inflammatory processes in different pathologies. However, the mechanism involved in the A_2A_ R-mediated increase in pro-inflammatory cytokine expression in the brain has remained elusive. Several recent studies have indicated that CF was expressed in activated microglial cells, and CF expression was substantially up-regulated in regions of white matter rarefaction in various demyelinating diseases of the central nervous system^7^. Consistent with previous data, our *in vitro* experiment also showed that CF was highly expressed in activated microglial cells in white matter lesions, which were induced by chronic cerebral hypoperfusion^9^. The previously described results suggested that up-regulated CF expression may be associated with the exacerbation of white matter lesions after cerebral hypoperfusion-induced brain injury and other demyelinating diseases.

Numerous studies have supported a key role for CF in various types of inflammation. For example, CF expression is primarily limited to immune cells, such as T cells, NK cells and dendritic cells, and its mRNA level is significantly up-regulated during the maturation and activation of immune cells from quiescent precursors^23^. CF modulates specific immune responses by inhibiting lysosomal cathepsin, which plays a role in immune cell activation and the production of pro-inflammatory cytokines and adhesion factors^24,25^. Recently, CF was identified as an endogenous inhibitor of cathepsin C, which plays a vital role in the production of inflammatory cytokines^20^. Thus, CF exerts a pro-inflammatory effect on the neuroinflammation in brain injuries by activating inflammatory cells and promoting the production of pro-inflammatory cytokines. More importantly, we have previously shown that A_2A_ R deficiency up-regulated CF expression, whereas A_2A_ R activation down-regulated CF expression in white matter lesions after chronic cerebral hypoperfusion. The results from our previous study have suggested that increased CF expression may participate in A_2A_ R-mediated modulation of inflammation in chronic cerebral hypoperfusion-induced white matter lesions^9^. Consistent with the previous results, the current findings also showed that A_2A_ R activation by CGS21680 significantly promoted CF mRNA and protein levels in BV2 cells following low glucose and hypoxia treatments. These results showed that the elevated expression of CF in activated BV2 cells is likely related to the A_2A_ R activation-induced modulation of inflammation in low glucose conditions and hypoxia-related diseases. Furthermore, we also found that A_2A_ R inactivation by SCH58261 markedly inhibited the CF mRNA levels in BV2 cells following low glucose and hypoxia treatments. However, SCH58261 significantly increased the protein levels of the CF dimer and CF monomer in BV2 cells at 8 h after the cells were exposed to low glucose and hypoxia. These results indicated that the protein expression of CF in ischemic BV2 cells was regulated by factors other than adenosine A_2A_ R. Since CF was shown to be secreted as a disulfide-linked dimer, which is inactive until it is reduced to its monomeric form^13^, its activity and the balance between the two forms of CF can be regulated. Further studies of the mechanisms of CF protein expression will be performed.

To further confirm the previous hypothesis, we blocked CF gene expression in BV2 cells with a CF shRNA viral vector prior to A_2A_ R activation or inactivation. The current results showed that the mRNA and protein levels of IL-1β, IL-6 and TNF-α, particularly the IL-1β and IL-6 expression, in the shRNA vector + CGS group were significantly decreased compared with the control vector + CGS group after the low glucose and hypoxia treatments. These results indicated that CF gene knockdown in hypoxic BV2 cells substantially inhibited the increase in the expression of A_2A_ R activation-induced pro-inflammatory mediators, such as IL-1β, IL-6 and TNF-α. Moreover, these results suggested that elevated CF expression may be involved in A_2A_ R-mediated modulation of neuroinflammation, which has been associated with activated microglial cells and increased expression of pro-inflammatory cytokines in ischemic brain injuries. More importantly, we also found that there was a difference between the mRNA level and protein expression of inflammatory cytokines. The mRNA level of IL-6 in the shRNA vector + SCH group was significantly decreased compared with that in the control vector + SCH group at 12 h after the cells were exposed to low glucose and hypoxia (*p* < 0.05). However, there was no difference in the protein expression of IL-6 between the shRNA vector + SCH group and the control vector + CGS/SCH group at 12 h after the cells were exposed to low glucose and hypoxia. Moreover, the mRNA levels of IL-1β and TNF-α in the shRNA vector + CGS/SCH group were significantly decreased compared with those in the control vector + CGS/SCH group at 4 h after the cells were exposed to low glucose and hypoxia (*p* < 0.05). However, there was no difference in the protein expression of IL-1β or TNF-α between the shRNA vector + CGS/SCH group and the control vector + CGS/SCH group at 4 h after the cells were exposed to low glucose and hypoxia. These results indicated that the mRNA and protein expression of inflammatory cytokines may be regulated by factors other than CF and A_2A_ R.

We have shown that CF plays an important role in modulating neuroinflammation induced by A_2A_ R activation in hypoxic BV2 cells. However, the underlying mechanisms involved in the effect of A_2A_ R activation on the CF expression under hypoxic conditions remain to be elucidated. CF expressed in inflammatory cells regulates the activation of inflammatory cells and the production of inflammatory cytokines^26^. A previous study indicated that CF is synthesized as an inactive dimer and is converted to the active monomeric form by proteolysis^13^. However, the identities of the factors responsible for the induction of CF expression in BV2 cells in response to hypoxia have remained elusive. As the adenosine A_2A_ R is a member of the G-coupled receptor family^27,28^, it signaling pathway activation is mediated by Gs protein-dependent adenylate cyclase (AC) activation, which leads to an increase in intracellular cAMP concentrations^29^, and the subsequent activation of PKA by cAMP may result in the phosphorylation of CRE binding protein (CREB), a nuclear transcription factor^30^. In addition to the cAMP/PKA/CREB signaling pathway, stimulation of the A_2A_ R results in the activation of the ERK signaling cascade through numerous different mechanisms that depend on the cell type^2^. For example, Gs protein stimulation results in PKA-mediated activation of ERK1/2 in tartrate-resistant acid phosphatase-positive (TRAP+) multinucleated cells that express the A_2A_ R^31^. Furthermore, the ERK signaling pathway is activated by PKC, which is activated by the A_2A_ R^32^. In addition, the JAK/STAT signaling network exerts an anti-inflammatory effect by regulating the production of anti-inflammatory cytokines associated with A_2A_ R-induced regulation of inflammation following brain injury^33^.

In the present study, we used the PKA inhibitor H-89, the PKC inhibitor staurosporine and the JAK inhibitor AG490 to interrupt the corresponding signaling pathways prior to A_2A_ R activation; we subsequently examined the CF mRNA and protein levels in hypoxic BV2 cells using RT-PCR, western blotting and immunofluorescence staining after the low glucose and hypoxia treatments to further investigate the signaling pathways involved in regulating CF expression following A_2A_ R activation. The current results showed that the CF mRNA levels in the A_2A_ R-activated BV2 cells were significantly reduced in the H-89 and staurosporine groups after the low glucose and hypoxia treatments compared with those in the VEH group. Consistent with the mRNA analysis results, the protein expression of the CF monomer in A_2A_ R-activated BV2 cells was substantially reduced in the H-89 and staurosporine groups compared with that in the VEH group, as detected by western blot and immunofluorescence staining; these findings indicate that both the PKA inhibitor H-89 and the PKC inhibitor staurosporine blocked the CGS21680-induced increased expression and activation of CF in BV2 cells after the low glucose and hypoxia treatments. Furthermore, we determined that the phosphorylation of CREB in the H-89 and staurosporine groups of A_2A_ R-activated BV2 cells was significantly decreased and the phosphorylation of ERK1/2 in the staurosporine group of A_2A_ R-activated BV2 cells was substantially decreased compared with that in the VEH group after the low glucose and hypoxia treatments. Therefore, these results suggested that the PKA/CREB and PKC/CREB or ERK1/2 signaling pathways were associated with the A_2A_ R-mediated up-regulation of CF expression in BV2 cells after the low glucose and hypoxia treatments. The present data may help shed light on the underlying mechanisms of the A_2A_ R activation-mediated neuroinflammatory response in neonatal hypoxic injuries and indicate that microglia A_2A_ R inactivation represents a potential therapeutic target for neonatal hypoxic brain injuries.

## Materials and Methods

### BV2 microglia cell culture

The murine microglia cell line BV2 were purchased from the American Type Culture Collection (Manassas, VA) and cultured in Dulbecco’s Modified Eagle’s Medium (DMEM)/F12 supplemented with 10% fetal bovine serum (FBS) (Thermo Scientific Hyclone, Logan, UT, USA), 2 mM glutamine, 100 U/ml penicillin and 100 mg/ml streptomycin. All cell lines mentioned previously were kept at 37 °C in a humidified atmosphere containing 5% CO_2_.

### Reagents and Antibodies

Adenosine A_2A_ R agonist CGS21680 and antagonist SCH58261 were purchased from Tocris (Bioscience, Bristol, UK); PKA inhibitor H-89, PKC inhibitor staurosporine and JAK inhibitor AG490 were purchased from Sigma-Aldrich (St Louis, MO, USA); Antibiotics (10,000 U/mL penicillin and 10,000 μg/mL streptomycin), 2.5% trypsin (10×), fetal bovine serum (FBS), Dulbecco’s Modified Eagle Medium (DMEM), DMEM: nutrient mixture F-12 (DMEM/F-12) and Hanks Balanced Salt Solution (HBSS) were purchased from Life Technologies Corporation (Grand Island, NY, USA). CF antibody was purchased from Santa Cruz (Santa Cruz Biotechnology, Inc. CA); TNF-α, IL-1β, IL-6 antibodys were purchased from Abcam (Abcam, Cambridge, UK); CREB/p-CREB antibody and ERK/p-ERK antibody were purchased from Cell Signaling Technology (Beverly, MA); STAT3/p-STAT3 antibody was purchased from Sigma-Aldrich (Sigma-Aldrich, St Louis, MO, USA).

### CF shRNA viral vector construction and transfection

To exclusively knockdown CF the following oligonucleotides were designed: 5′-gatccgtgccctcctctccagattaacttcaagagagttaatctggagaggagggcactttttg-3′ and 5′-aattcaaaaagtgccctcctctccagattaactctcttgaagttaatctggagaggagggcacg-3′. CF shRNA was constructed from Dharmacon Inc. (Lafayette, CO) and used to target mice CF (GenBank Accession Number: NM_009977). BV2 microglial cells were transfected with CF shRNA viral vector (50 nM) by using the DharmaFECT shRNA transfection reagent according to the manufacturer’s instructions. Nonspecific shRNA (Dharmacon Inc.) that does not target any mouse genes was used as a negative control. Briefly, after subculture, BV2 cells were plated in 12-well plates at a density of 3 × 10^4^ cells/ml. This was followed by adding 250 ul CF shRNA vector and control vector. The cells were incubated with the shRNA mix for 24 h and then the medium was replaced with DMEM with 2% FBS without antibiotics and plated in 6-well plates. Then, the cells were incubated with the shRNA vector or control vector for another 24 h and then the medium was replaced with DMEM with 2% FBS without antibiotics and incubated for another 24 h for RNA and protein extraction to check the knockdown efficiency by reverse transcription (RT)-PCR and western blot analysis. At the same time, the cell viability of CF shRNA vector or control vector transfected cells and control cells was assessed by a tetrazolium salt (WST-8)-based colorimetric assay in the Cell Counting Kit 8 (Dojindo, Kumamoto, Japan)^34^. Briefly, CF shRNA vector or control vector transfected cells and control cells were seeded onto 96-well plates at an initial density of 5 × 10^3^ cells/well. At specified time points, 10 µl of CCK-8 solution was added to each well of the plate. Then the plate was incubated for 4 h. Cell viability was determined by scanning with a microplate reader at 450 nm. Data were expressed as the percentage of viable cells as follows: relative viability (%) = [OD450 (transfected) − OD450(blank)]/[OD450(control) − OD450(blank)] × 100%.

### *In vitro* model of ischemia and examination of cell viability

The culture medium was removed and BV2 microglial cells were washed twice with glucose-free DMEM (Invitrogen). BV2 microglial cells were then switched from a normal feeding medium to a low glucose DMEM/F12medium (1 g/L) in an hypoxia incubator (3% O_2_, 5% CO_2_, 92% N_2_) for 4 h, 8 h and 12 h^35,36^. The cell viability was assessed by a tetrazolium salt (WST-8)-based colorimetric assay in the Cell Counting Kit 8 (CCK-8; Dojindo, Kumamoto, Japan)^34^. Data were expressed as the percentage of viable cells as follows: relative viability (%) = [OD450 (ischemia) − OD450(blank)]/[OD450(control) − OD450(blank)] ×100%.

### Pharmacological treatments

To investigate the role of CF in the inflammatory modulation induced by A_2A_ R in hypoxic-BV2 cells, we transfected BV2 cells with CF-targeting shRNA viral vectors and control viral vectors. Then, we added the A_2A_ R agonist CGS21680 (100 nM)^22^ or A_2A_ R antagonist SCH58261 (50 nM)^21,37^ into the CF shRNA transfected BV2 cells and the control vector transfected BV2 cells at 10 min before incubation in an low glucose DMEM/F12 medium (1 g/L) in an hypoxia incubator (3% O_2_, 5% CO_2_, 92% N_2_) for 4 h, 8 h and 12 h to activate or inactivate of adenosine A_2A_ R in BV2 cells. Then, the mRNA levels and protein expressions of CF, TNF-α, IL-1β and IL-6 in hypoxic-BV2 cells was also detected by RT-PCR, Western Blot and ELISA at 4 h, 8 h and 12 h after the hypoxia and low-glucose incubation. To elucidate the signaling pathways associated with how A_2A_ R acted on CF expression in hypoxic-BV2 cells, we added 5 μM PKA inhibitor, H-89^38^ or 100 nM PKC inhibitor, stauroporine^39,40^ or 50 μM JAK inhibitor, AG490^41^ respectively to the BV2 cells at 30 min before the hypoxia and low-glucose incubation, followed by the treatment of CGS21680 at 10 min before the hypoxia and low-glucose incubation. At these doses, H-89, stauroporine or AG490 can exert the inhibitory effect on PKA, PKC or JAK substrate phosphorylation and related cellular functions. And then, the mRNA level and protein expression of CF was detected by RT-PCR, western blot analysis and immunofluorescence staining at 8 h after the hypoxia and low-glucose incubation. Furthermore, the protein expression of the phosphorylated substrate proteins in the PKA, PKC and JAK pathways was also examined by western blot analysis.

### RNA extraction and quantitative real-time reverse transcription PCR analysis

BV2 cells were grown in 10 cm dishes and washed once with PBS, and total RNA was extracted using TRIzol reagent (Invitrogen, Carlsbad, CA) according to the manufacturer’s protocol. RT reactions were performed using the RT system kit (Promega, Singapore). The RNA concentration and quality were evaluated spectrophotometrically. The total RNA was used to reverse transcribe cDNA. The resultant cDNA was diluted 10 times in double distilled H_2_O and kept at −20 °C for RT-PCR analysis. The primers for CF, inflammatory cytokines and GAPDH are shown in Table 1. RT-PCR was performed using a Light-Cycler (Roche Diagnostics, Indianapolis, IN, USA), and individual RT-PCRs were carried out in glass Light Cycler capillaries (Roche Diagnostics) according to the manufacturer’s instructions. The RT-PCRs were carried out in a 10 μl final volume containing the following: 5 μl SYBR Green I master mix Qiagen); 1 μl of 5 μM forward primer and 1 μl of 5 μM reverse primer; and 3 μl of diluted cDNA. After an initial denaturation step at 95 °C for 4 min, temperature cycling was initiated. Each cycle consisted of denaturation at 95 °C for 40 sec, annealing at 60 °C for 40 sec, and elongation at 72 °C for 60 sec. In total, 40 cycles were performed. Mouse GAPDH was amplified as the control for normalizing the quantities of transcripts of each of the genes mentioned above. The results of the PCR analyses were confirmed in at least three independent experiments.

**Table 1:** Sequence of specific primers used for quantitative real-time PCR.

	Sense Primer (5′-3′)	Antisense Primer (5′ -3′)
CF	ACCAATAACCCAGGAGTGCTT	TGACCCAGACTTCAGAGTAGC
IL-1β	ACTGTTTCTAATGCCTTCCC	ATGGTTTCTTGTGACCCTGA
IL-6	GTTGCCTTCTTGGGACTGATG	ACTCTTTTCTCATTTCCACGATTT
TNF-α	CCTCTTCCCTGTCGCTAACTC	TAAACGTCTTTCTCCAGCTCC
GAPDH	ACCCATCACCATCTTCCAGGAG	GAAGGGGCGGAGATGATGAC

### Western blot analysis

Culture medium was removed from the culture plate, and BV2 cells were washed twice with ice-cold PBS. Then, the cells were washed with PBS and lysed in 20 mM Tris–HCl buffer (pH 7.4) containing a protease inhibitor mixture (0.1 mM phenylmethanesulfonyl fluoride, 5 mg/mL aprotinin, 5 mg/mL pepstatin A, and 1 mg/mL chymostatin). Protein concentration of samples was then determined by using a protein assay kit (Bio-Rad, Hercules, CA, USA, catalogue number 500–0002). Next, 30 μg of the protein sample was loaded and separated on 12% sodium dodecyl sulfate-polyacrylamide gels electrophoresis (SDS-PAGE). The proteins embedded in the gel were then transferred to polyvinylidene difluoride membranes using a semidry electrophoretic transfer cell (Bio-Rad). The membranes were washed with TBS-0.1% Tween buffer and then incubated with 5% nonfat dry skimmed milk for 30 min at room temperature. Next, they were respectively incubated with the primary antibody rabbit anti-TNF-α (1:1000; Abcam, Cambridge, UK)), rabbit anti-IL-1β (1:1000; Abcam, Cambridge, UK), rabbit anti IL-6 (1:1000; Abcam, Cambridge, UK), and rabbit anti GAPDH (1:1000; Sigma-Aldrich, St Louis, MO, USA), rabbit anti-CREB/p-CREB and rabbit anti-ERK/p-ERK (1:800; Cell Signaling Technology, Beverly, MA), goat anti-STAT3/p-STAT3 (1:800; Sigma-Aldrich, St Louis, MO, USA) overnight on a shaker at 4 °C. After three washes with TBS-0.1% Tween, the membranes were incubated with horseradish peroxidaseconjugated secondary antibody for 1 h. The proteins were detected with a chemiluminescence detection system according to the manufacturer’s instruction (Supersignal West Pico Horseradish Peroxidase Detection Kit; Pierce Biotechnology, Rockford, IL, USA, catalogue number 34077) and developed on the film. The optical densities (OD) of specific electrophoretic protein bands were scanned and measured using image analysis software (Quantity One 4.4.0.36, USA) and normalized to GAPDH. The protein expression of CF was detected by nonreducing SDS-PAGE gel analysis using the primary antibody goat anti-CF (1:300; Santa Cruz Biotechnology). The OD of specific electrophoretic protein bands were scanned and normalized to Tubulin. The above results of the western blot analysis were confirmed in at least three independent experiments.

### Enzyme-linked immunosorbent assay (ELISA)

ELISA was performed as per the manufacturer’s instructions (Dakewe Biotech, Shenzhen, China) to assess the concentrations of TNF-α, IL-1β and IL-6 in the BV2 cells culture supernatant. The results of the ELISA were confirmed in at least three independent experiments.

### Immunofluorescence staining in BV2 cells

BV2 cells were fixed with 4% paraformaldehyde in 0.1 M PBS for 15 min. Following rinsing with PBS, the coverslips with adherent cells were used for immunofluorescence staining. In every group, BV-2 cells were incubated with goat anti-CF antibody (1:100; Santa Cruz Biotechnology) overnight at room temperature. Subsequently, the cells were incubated in FITC-conjugated secondary antibodies for 1 h at room temperature. After washing, 4′,6-diamidino-2-phenylindole (DAPI, 10 ug/ml, Beyotime, China) was used to counterstain the cell nuclei. The fluorescent sections were observed and photographed using a confocal laser-scanning microscope (TCSTIV; Leica, ussloch, Germany). To evaluate the immunofluorescence staining, a semi-quantitative analysis was performed as previously reported^42^, integral optical density of immunopositive cells and region of interest were measured by Image-Pro Plus 6.0 image analysis software (Media Cybernetics Inc, USA). Values (five slides for every group) of optical density in individual cells slide represented quantities of objective proteins. For quantification, mean optical density is the medium value of optical density in 3 to 5 interest areas, which were captured for each slide of every group. In this study, an interest area represents an invariable dimension.

### Statistical analysis

Data are expressed as mean ± SEM. For evaluation of CF expression in BV2 cells, Statistical comparisons were carried out using a one-way analysis of variance (ANOVA) followed by Tukey’s Multiple Comparisons Test. Quantitative-PCR, western blot and ELISA data were analyzed using a two-way analysis of variance followed by Student-Newman-Keuls test for multiple comparisons. Statistical analyses were performed using SPSS for Windows (SPSS 15.0; SPSS Inc, Chicago, IL). The minimal level of significance was identified at *p* < 0.05.
